# The Anti-Vivisection Societies and the Hospitals

**Published:** 1895-11-30

**Authors:** B. Burford Rawlings

**Affiliations:** Secretary to the National Hospital for the Paralysed and Epileptic.


### THE ANTI-VIVISECTION SOCIETIES AND THE HOSPITALS.

By B. Burford Rawlings, Secretary to the National Hospital for the Paralysed and Epileptic.

We reprint the following interesting article from the *Times* of Wednesday, 27th inst.

"Some time ago you allowed me, in view of the injury which misapprehension, fostered by the anti-vivisection societies, was inflicting upon some if not all hospitals, to put forward in your columns a statement to the effect that hospitals are in no way concerned in the practice of vivisection; that no act of vivisection, so called, has ever taken place in an English hospital; that the hospital authorities have never been called upon to express an opinion for or against the practice; that they possess no power either to promote or hinder it; and that, in short, they are as free from all responsibility in respect of it as its most conscientious opponent.

The accuracy of that statement was not challenged. Yet the representatives of the societies have continued to denounce in their prints one and another of the hospitals, and to do their utmost to prevent subscriptions reaching them, for no better reason than that the physicians and surgeons who happen to be members of the staff see fit to exercise in their private capacity a personal liberty of thought and action in reference to a matter the societies feel strongly about, but with which the hospitals have no concern.

I am not desirous, nor would it be profitable for me, to say one word upon the merits of the controversy, of which most people are weary. On the contrary, my aim is to advance nothing but what might be consistently urged by the fair-minded of both parties. Let vivisection be all its advocates or all its assailants aver, the fact tells neither for nor against the hospitals. The merit or the blame is not theirs.

"That the charges made by the representatives of the societies as they affect the hospitals are unwarranted and libellous no one with a knowledge of the subject can doubt, and that these libels are effective and damaging is too true. Dr. Wilks has lately reminded us in the *Times* that the public do not support hospitals. The maintenance of our unendowed medical institutions is almost wholly the work of a mere handful of generous men and women, and in all probability there is not a hospital in the metropolis dependent upon contributions but is indebted, in any given year, for the larger part of its receipts to the benefactions of less than a dozen individuals. It is manifest, therefore, how easily the attention of hospital supporters may be intruded upon, and how serious the injury is though the sympathies of only one of these good friends be arrested.

It would be easy to name some specific instances of harm suffered—three came under my notice lately within the space of a few days—although, of course, much of it can never be actually demonstrated. Quite recently your columns contained a list of bequests to societies amounting to some £60,000, of which the large majority were for the benefit of animals other than man—horses, dogs, &c.—while the institutions which are supreme in their beneficence and utility were conspicuously absent. The explanation was found in a legacy of large amount bequeathed to an 'anti-vivisection society,' and, without for a moment seeking to lessen the full liberty of a testator, hospital workers may be permitted a protest when they believe the exclusion of their institutions is brought about by reckless misstatement. The animus exhibited in the provisions of this particular will is remarkable chiefly because of its increasing frequency, and we may well ask, What is the result these assailants of our hospitals have set themselves to achieve?

"They would probably tell us that their efforts are directed to purge the hospitals of physicians and surgeons who favour vivisection. That is, they desire to compel the authorities to appoint none but its opponents to places upon the staff. But that is impossible, seeing that the profession, rightly or wrongly, is practically unanimous; and assuming that any body of governors or managers were to resolve upon the action the societies desire, and could insist, which is doubtful, the consequence must be the closing of the hospital, and if the closing of one hospital, then eventually of all hospitals, as if any are involved then all.

"Is that a result the many benevolent and scrupulous supporters of the societies are prepared to view with satisfaction and to assist in promoting? Already, no doubt without realising it, they are being led to take the heavy responsibility of lessening the power of hospitals to perform their legitimate work. They are withholding tendance from and inflicting needless suffering upon some among the sick who depend upon the hospitals for relief. To shut the door upon stricken men and women is a terrible act, even if they are sinners deserving punishment, but if they are only helpless and innocent the wrong done is immeasurable, and it is one no after act can repair.

"Then, supposing, what is certainly not the case, that the pure philanthrophy of our hospitals—that is, their work among the poor—is accounted as nothing by the kindly people who are persuaded into hostility towards it, what is to be said of the attitude, not less selfish than illogical, into which their withdrawal of help from the hospitals betrays them? To fully appreciate the position we must recall the elementary truth, that the beneficence of our hospitals is shared by all classes, that hospitals are the sources of all medical and surgical knowledge, and the schools of nursing. There is no reason to suppose that the supporters of anti-vivisection societies, when ill, decline the services of a doctor or a nurse, and it follows therefore that they are willing to make use of means for their own relief which they reprobate elsewhere, and that they allow their sentiment so to run away with their reason as to punish a patient, other than themselves, for the sins they allege against the doctor.

"Again, persons who refuse support to the hospital because the members of its staff are under the ban of the anti-vivisection societies are not always averse from sending their nominees for treatment. They only object to subscribe. The hospital is not worthy to be supported, but that is no reason why it should not be made use of.

"And this is not all. If an anti-vivisectionist fall ill in the street, or become the victim of an accident, as a matter of course he is carried to the nearest hospital, and in all likelihood, if conscious, he would demand to be taken there, well assured of receiving skilful and tender treatment. To complete the picture, we must have outside all his fellow-thinkers who are not in similar case occupied in execrating the hospital, banded together in a conspiracy to destroy it, and, to the extent that he is dependent upon the hospital, to destroy him with it.

"The force of unreason, it may be almost said, of barbarity, could scarcely go further. The cause of the societies may be wholly righteous, but let it be asserted by fair means. At present the chief exploits of these organisations are connected with attacks upon the hospitals, where lie the sick and maimed of both sides, but chiefly of no side. For the sake of humanity and in the name of our civilisation, is it not time that this inhuman war shall be made to cease?"

